# Design Procedure and Fabrication of Reproducible Silicon Vernier Devices for High-Performance Refractive Index Sensing

**DOI:** 10.3390/s150613548

**Published:** 2015-06-10

**Authors:** Benedetto Troia, Ali Z. Khokhar, Milos Nedeljkovic, Scott A. Reynolds, Youfang Hu, Goran Z. Mashanovich, Vittorio M. N. Passaro

**Affiliations:** 1 Department of Electrical and Information Engineering, Politecnico di Bari, Via E. Orabona 4, 70125 Bari, Italy; E-Mail: benedetto.troia@poliba.it; 2 Optoelectronics Research Centre, University of Southampton, Southampton SO17 1BJ, UK; E-Mails: a.z.khokhar@soton.ac.uk (A.Z.K.); m.nedeljkovic@soton.ac.uk (M.N.); s.reynolds@soton.ac.uk (S.A.R.); y.hu@soton.ac.uk (Y.H.); g.mashanovich@soton.ac.uk (G.Z.M.)

**Keywords:** integrated waveguide sensors, optical sensing, Vernier effect, silicon

## Abstract

In this paper, we propose a generalized procedure for the design of integrated Vernier devices for high performance chemical and biochemical sensing. In particular, we demonstrate the accurate control of the most critical design and fabrication parameters of silicon-on-insulator cascade-coupled racetrack resonators operating in the second regime of the Vernier effect, around 1.55 μm. The experimental implementation of our design strategies has allowed a rigorous and reliable investigation of the influence of racetrack resonator and directional coupler dimensions as well as of waveguide process variability on the operation of Vernier devices. Figures of merit of our Vernier architectures have been measured experimentally, evidencing a high reproducibility and a very good agreement with the theoretical predictions, as also confirmed by relative errors even lower than 1%. Finally, a Vernier gain as high as 30.3, average insertion loss of 2.1 dB and extinction ratio up to 30 dB have been achieved.

## 1. Introduction

The Vernier effect has been widely used in integrated photonics for filtering and sensing applications. Actually, silicon-on-insulator (SOI) ring and racetrack resonators (RRs) operating in the first regime of the Vernier effect have been designed and fabricated for enlarging and even eliminating the overall free spectral range (FSR) in linear optical filters [1,2], realizing tunable optical filters [3], reconfigurable optical switches [4], as well as widely tunable ring lasers [5,6].

In the context of integrated photonic refractive index (RI) sensors, thus based on the homogeneous or surface sensing principles [7], the second regime of the Vernier effect has been demonstrated to be particularly suitable for enhancing sensing performance, e.g., wavelength sensitivity,$\;S_{\lambda }$, and limit of detection (LOD) [8]. Actually, the Vernier effect has been demonstrated experimentally by cascading two coupled ring/racetrack resonators [9,10,11,12,13,14,15], two Mach-Zehnder interferometers (MZI) [16,17], or a mixed combination of them [18]. In particular, one of the two cascade-coupled devices is isolated in order to make one RR or MZI sensible to the chemical/biochemical specie to be sensed with the remaining device insensible to the surrounding environment. To this purpose, some experimental results of Vernier RI sensors reported in literature are listed in Table 1, specifying the corresponding sensing performance, the technology platforms employed as well as the waveguides and the device architectures designed.

**Table 1:** Fabricated silicon Vernier RI sensors operating in the near-infrared, around λ = 1.55 µm.

Ref.	Platform	Waveguide	Architecture	$S_{\lambda }$	LOD
*Homogeneous sensing*					
[10]	SOI	Rib	Cascade-coupled RRs	1.30 µm/RIU	5 × 10^−4^ RIU
[11]	SOI	Suspended nanowire	Cascade-coupled RRs	460 µm/RIU	4.8 × 10^−6^ RIU
[12]	SOI	Nanowire	Cascade-coupled RRs	24.30 µm/RIU	<7 × 10^−4^ RIU
[13]	SOI	Nanowire	Cascade-coupled RRs	2.17 µm/RIU	8.3 × 10^−6^ RIU
[14]	SOI	Nanowire	Cascade-coupled RRs	1.07 µm/RIU	1.6 × 10^−5^ RIU
[15]	SiN	Ridge	Cascade-coupled RRs	* 9.80 µm/RIU	* 2.0 × 10^−6^ RIU
[18]	SOI	Nanowire	Cascaded RR and MZI	21.5 µm/RIU	<2.3 × 10^−6^ RIU
*Surface sensing*					
[17]	SiN	Slot and strip	Cascaded MZIs	60 nm/(ng/mm^2^)	0.155 (pg/mm^2^)

* estimated theoretical sensing performance.

Actually, the approaches commonly used in order to enhance sensitivities and LODs of integrated photonic RI sensors have concerned with the design of highly performant integrated silicon-based waveguides, such as slot [19], membrane [20], or suspended [21] structures, by which the optical field confinement in the low RI medium, *i.e.*, the region where the specie to be detected is concentrated, can be enhanced. Consequently, the overlap between the propagating optical field and the analyte can also be maximized. Furthermore, the optimized design of integrated architectures such as resonant microcavities, photonic crystals, directional couplers (DCs) as well as MZIs [22,23,24], combined with selective chemical and biochemical surface functionalization techniques and advanced microfluidic systems has led to the experimental demonstration of CMOS-compatible ultra-high performance sensing platforms, which are suitable for label-free detection, high-throughput analysis, on-chip array integration as well as low cost and large-scale fabrication [24,25]. In this context, silicon photonic crystal nanobeam cavities are worth being mentioned, since they have been demonstrated as very efficient optical sensors. Indeed, a LOD as low as parts-per-billion (ppb) in ambient conditions has been measured [26] and the capability of heat supply in such devices has been successfully demonstrated for local temperature control in biochemical sensing applications [27].

Nowadays, the trend in the evolution of innovative RI photonic sensors consists, for example, in extending the operation of silicon photonics from the conventional near-infrared (NIR) to the vibrant mid-infrared (MIR) wavelength range, since a large number of chemical and biochemical specie as well as harmful gases exhibit very strong absorption lines in the “fingerprint region” of 8–16 µm [28,29]. Moreover, a further approach consists in designing complex and multi-device systems where a very accurate control of the design and fabrication parameters is required for achieving a very high reproducibility. Consequently, the development of novel lab-on-a-chip platforms allows the performance of laboratory functionalities by means of sensor matrices fabricated on the same chip [30,31,32]. In this context, the need of robust and reliable design tools is crucial to carry out cutting-edge research in these scientific areas.

In this paper, we propose a generalized procedure based on sophisticated algorithmic routines and rigorous mathematical background for the reproducible design of silicon Vernier devices characterized by cascade-coupled RRs. In fact, it is worth noting that in the case of both the first and second operating Vernier regimes, performance of Vernier devices depends dramatically on waveguide dimensions, RR lengths and power coupling coefficients of integrated DCs. Consequently, a rigorous and reliable parameter control is required in order to satisfy the expected device performance, ensure the proper device operation as well as achieve a high reproducibility in the design and fabrication of such devices. Finally, although a very accurate theoretical analysis of the influence of waveguide fabrication tolerances on DCs and single RRs has already been reported in literature [33], a similar approach with the evidence of the criteria used for the design of Vernier cascaded RRs have never been demonstrated experimentally. In fact, sensing performance and device operations are well demonstrated by means of experimental characterizations in the pioneering works reported in Table 1, but design criteria used for setting RR lengths as well as the DC dimensions are not reported at all, neither their influence on device performance which we consider worth being investigated. The only exception is represented by [15], where a number of experimental Vernier spectra are shown as a function of different values of the DC straight section length.

The overall design flow reported in this investigation has been tested on SOI Vernier devices characterized by cascade-coupled RRs based on silicon rib waveguides and working in the NIR wavelength range of 1520–1580 nm. Finally, the experimental evidence of the accuracy and reliability of the specific strategies developed by us for the design of Vernier devices operating in the second regime [34] as well as for the modelling of integrated photonic waveguides and DCs [35], is also demonstrated.

## 2. Design Strategies and Device Fabrication

**Figure 1:**
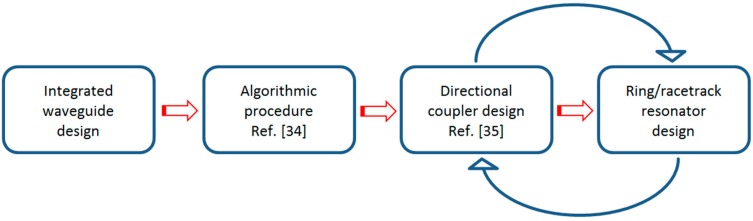
Diagram of the design flow for Vernier devices based on cascade-coupled ring/racetrack resonators for high-performance refractive index sensing.

The overall design flow of Vernier devices is sketched in Figure 1 and starts with the selection and design of the waveguides to be used for performing the optical signal propagation throughout the overall chip as well as the sensing functionalities into the sensible area. To this purpose, several strategies can be taken into account such as the design and fabrication of two different guiding structures to be used for the isolated and sensible RRs, respectively. For example, a designer can decide to use slot waveguides optimized for homogeneous or surface sensing into the sensible area and conventional strip/rib waveguides for the rest of the chip [17,36]. Similarly, the reference RR of the Vernier device can be based on standard silicon nanowire while the sensing RR based on suspended silicon nanowires with an homogeneous sensitivity $S_{h}=\;\text{Δ}n_{eff}/\text{Δ}n_{c}$ even higher than 1, with $\text{Δ}n_{eff}$ the variation of the effective index of the propagating optical mode induced by Δ$n_{c}$, *i.e.*, the variation of the cover medium RI into the sensible area [11].

The waveguide design procedure takes into account the polarization of the propagating optical signal as well as the different cover media surrounding the waveguides. Indeed, a cover layer is usually used for isolating a typical Vernier architecture where a sensible window is then opened on one of the two cascaded devices (e.g., RRs, MZIs), so that it can be exposed to the analyte to be sensed. For example, SU8 polymer [10,12,18], polydimethylsiloxane (PDMS) [17], benzocyclobutene-based polymer [14] as well as silicon oxide [11,13] have been used to this purpose in Vernier chemical and biochemical sensors operating in the NIR wavelength range, particularly around 1.55 µm. As a result, the optical properties of the reference and sensing device waveguides, such as the group index, $n_{g}$, as well as the effective index, $n_{eff}$, will be different, so resulting in different operation of the cascade-coupled devices.

In this investigation, conventional SOI rib waveguides have been selected for the design and fabrication of Vernier architectures based on cascade-coupled RRs and operating in the NIR. Furthermore, it is worth specifying that, since the scope of this investigation is not related to the demonstration of a novel Vernier sensor but of a sophisticated design flow as a flexible and reliable tool for the reproducible design and fabrication of such devices, covering layers have not been taken into account in the waveguide design and fabrication so that both cascade-coupled RRs are covered by air.

**Figure 2:**
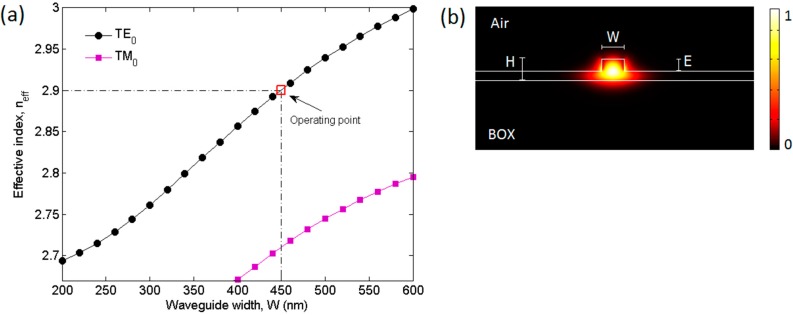
(**a**) Effective indexes of the fundamental quasi-TE and quasi-TM modes as a function of the waveguide width, *W*, with *E* = 220 nm and λ = 1.55 µm. The multimode cut-off width is *W* ≈ 650 nm; (**b**) $E_{x}$ optical mode spatial distribution in the SOI rib waveguide with nominal dimensions *W* = 450 nm, *E* = 220 nm operating at the NIR wavelength of 1.55 μm.

A 6-inch SOI wafer with a 400 nm-thick (*H*) silicon top layer and a buried oxide (BOX) thickness of 2000 nm has been used for the device fabrication and taken into account for simulations. Consequently, a typical waveguide mode analysis has been performed as shown in Figure 2a,b. In particular, the effective index of the fundamental quasi-TE (transverse electric) and TM (transverse magnetic) polarized optical modes are plotted as a function of the waveguide width (*W*) varied in the range of 200–600 nm with the etch depth (*E*) fixed at 220 nm (Figure 2a). Moreover, the fundamental quasi-TE-polarized optical mode spatial distribution has been simulated in the single-mode SOI rib waveguides characterized by the nominal dimensions:$\;W_{nom}$ = 450 nm and $E_{nom}$ = 220 nm (Figure 2b). With reference to Figure 2a, the operating point identified by the coordinates ($W_{nom}$,$\;E_{nom}$) indicates the excitation of the fundamental quasi-TM mode that has been prevented by means of a polarization controller in the experimental setup. In fact, the SOI rib waveguide patterns in a non-chemically amplified high resolution positive resist ZEP-520A have been written using a JEOL JBX 9300FS electron-beam (e-beam) lithography tool and transferred to the SOI wafers by inductively coupled plasma (ICP) etching.

**Figure 3:**
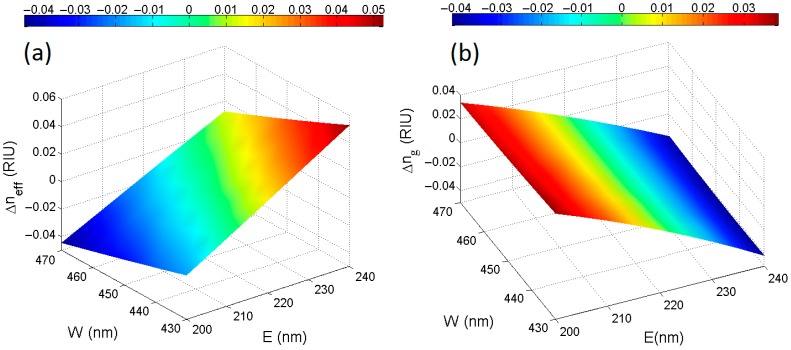
SOI rib waveguide quasi-TE optical mode (**a**) effective and (**b**) group index variations as a function of fabrication tolerances at the operating wavelength of 1.55 μm.

Once the waveguide structure has been selected, the design strategy consists in estimating theoretically the influence of process variability on Vernier device operation and performance as a function of the technology platform employed and the available foundry. To this purpose, fabrication tolerances for the SOI rib waveguide width and height of ±20 nm with respect to the nominal dimensions (*i.e*.,$\;W_{nom}$ = 450 nm, $E_{nom}$ = 220 nm) have been taken into account in the design procedure. In particular, rigorous simulations based on the two-dimensional (2D) full vectorial finite element method (FEM) [37] have been performed for calculating three-dimensional (3D) maps of waveguide effective and group indices, as plotted in Figure 3a,b, respectively.

Numerical results evidence that the most critical fabrication parameter is the etch depth *E* since the maximum index variations Δ$n_{eff|g}=n_{eff|g}\left(W_{nom},E_{nom}\right)-n_{eff|g}(W,E)$ as a function of *E* in the range 200–240 nm with *W* fixed at 450 nm, are as large as Δ$n_{eff,max}$ = −0.0318 RIU and Δ$n_{g,max}$ = −0.0415 RIU. On the contrary, the curves of Δ$n_{eff}$ and Δ$n_{g}$ as a function of *W* are almost flat, as observable in Figure 3a,b, respectively. In conclusion, it is worth noting that a similar investigation can be performed for any desired waveguide by means of special custom-made codes developed in FEMLAB platform [37] and adapted to the special guiding structures to be designed and fabricated, independently from the technology platform employed. Finally, chromatic dispersion is also take into account by means of Sellmeier equations for silicon [38] and its oxide [39,40].

With reference to Figure 1, the outputs of the block named as “Integrated waveguide design”, thus the 3D maps of the waveguide effective and group indices, are given as input parameters to the second step of the overall design flow, *i.e.*, the algorithmic procedure to be performed in order to find out proper cascade-coupled RR lengths to achieve the desired Vernier effect and sensing performance as a function of the specific analyte to be sensed [34]. In particular, an arbitrary set of four power coupling coefficients ${\text{κ}}_{c,i}^{2}$, thus one for each DC between a RR and the coupled bus waveguides as well as an initial set of RR lengths $L_{\#1}$ and $L_{\#2}$, are given as input parameters of the algorithmic procedure in addition to the aforementioned 3D maps of $n_{g}$ and $n_{eff}$. Specifically, the set of DC power coupling coefficients ${\text{κ}}_{c,1}^{2}=\;{\text{κ}}_{c,2}^{2}=\;{\text{κ}}_{c,3}^{2}=\;{\text{κ}}_{c,4}^{2}=0.1$ and the set of RR lengths ranging from 200 µm to 2500 µm in a 0.05 µm increment, have been processed in this investigation. Moreover, it is worth specifying that a detailed description of the algorithmic flow chart is reported and described in [34], so only the most significant numerical results of the algorithmic procedure are presented in this manuscript.

The Vernier effect in integrated photonic devices can be achieved by cascading two ring or racetrack resonators properly coupled to each other and designed with slightly different roundtrip lengths so that their FSRs (*i.e*., $FSR_{\#1}$ and $FSR_{\#2}$) are different as well. In this way, some resonant wavelengths of cascaded RR spectra will be overlapped while the remaining peaks will be misaligned, generating the typical comb-like Vernier spectrum. Furthermore, the overall Vernier transmittance is the product of single RR transmittances [9,10,11,12,13,14,15,16,17,18,34], but, depending on the relationship between the cascaded FSR difference, *i.e*., $∆FSR\;=\;\left|FSR_{\#1}-FSR_{\#2}\right|$, and the minimum linewidth at the full-width-at-half-maximum (FWHM) among the resonance peaks of both RRs, *i.e*., $\text{min}({\text{Δλ}}_{FWHM\left(\#1,\#2\right)})$, it is possible to select between the operation of Vernier devices in the first or second operation regime. In particular, Vernier devices designed and fabricated in this investigation operate in the second Vernier regime, thus Equation (1) is satisfied [13]:

(1) $$∆FSR<min\left(∆{\text{λ}}_{FWHM(\#1,\;\#2)}\right)$$

On the contrary, the first regime occurs when $∆FSR>min\left(∆{\text{λ}}_{FWHM(\#1,\;\#2)}\right)$. The overall Vernier FSR, *i.e*., $FSR_{v}$, defines the distance between two consecutive Vernier peaks and it can be calculated as $FSR_{v}=\;\left(FSR_{\#1}\times FSR_{\#2}\right)/∆FSR$. Consequently, the Vernier gain factor, *G*, is equal to the ratio between $FSR_{\#1}$ and ∆FSR and it allows to quantify the advantage of using such architectures over single RR or single MZI. For example, in the case of a RR used for RI sensing the wavelength shift induced by a cover RI change is $∆{\text{λ}}_{sensor}=\;{\text{λ}}_{sensor}\cdot (∆n_{eff}/n_{eff})\;$with ${\text{λ}}_{sensor}$ a resonant wavelength of the RR. Consequently, the wavelength sensitivity can be calculated by means of the equation $S_{\text{λ}}=\;(∆{\text{λ}}_{sensor}/\text{Δ}n_{eff})\cdot (∆n_{eff}/n_{c})$. As a result, it is possible to demonstrate both theoretically and experimentally [9,10,11,12,13,14,15,16,17,18,34] that in the case of Vernier devices based on cascade-coupled RRs the sensing performance aforementioned can be enhanced by the gain factor *G*, resulting in $∆{\text{λ}}_{v}=\;∆{\text{λ}}_{sensor}\cdot G$ and $S_{\text{λ},v}=\;S_{\text{λ}}\cdot G$. Finally, it is worth specifying that cascade-coupled RR lengths must be chosen as a function of the specific analyte to be sensed, being the minimum detectable RI change $∆n_{c,min}$ equal to $n_{eff}\cdot \;\left(∆FSR/{\text{λ}}_{sensor}\right)\cdot \left(∆n_{c}/∆n_{eff}\right)$ [34].

The application of the generalized algorithmic approach based on Mason’s rule and delay line signal processing in the *Z*-transform domain proposed by us and described in [34], has resulted in a total number of 140 possible combinations of cascade-coupled RR lengths suitable for achieving the Vernier effect in the second operating regime at the NIR wavelength range of 1520–1580 nm.

**Figure 4:**
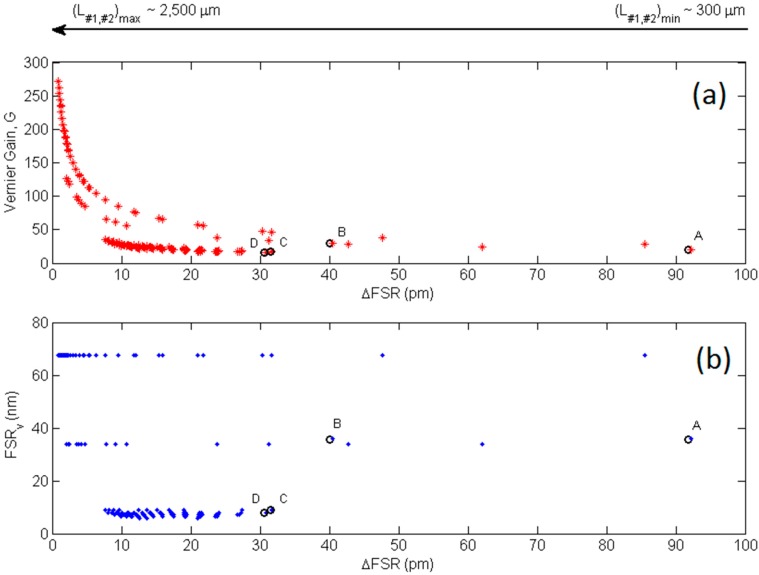
(**a**) Vernier gain *G* and (**b**) free spectral range, $FSR_{v}$, as a function of ∆FSR corresponding to the Vernier configurations resulted from the algorithmic procedure.

A graphical representation of the calculated algorithmic solutions is given in Figure 4a,b, where each of the 140 combinations is associated to the resulted ∆FSR and plotted with the corresponding Vernier gain, *G*, and overall Vernier FSR, $FSR_{v}$. Referring to Figure 4a, it is evident that the Vernier gain increases exponentially with decreasing the value of ∆FSR. Theoretically, gain *G* even higher than 250 can be achieved with ∆FSR as small as few pm. Furthermore, very small values of ∆FSR correspond to long cascade-coupled RR lengths (*i.e*., $L_{\#1}$, $L_{\#2}$), as indicated by the arrow at the top of the plot (Figure 4), and the number of the calculated combinations is not equally distributed along the ∆FSR vector since most of them are concentrated towards decreasing values of ∆FSR. This behavior is due because the longer the RR lengths the shorter the FSRs of the cascade-coupled RRs and the higher the number of RR resonances in the selected wavelength range of 1520–1580 nm, thus resulting in a more favorable condition for achieving the second regime of the Vernier effect, according to Equation (1). Finally, it is worth specifying that values of *G* and $FSR_{v}$, plotted in Figure 4a,b, respectively, correspond to different Vernier configurations with specific RR lengths. Moreover, the algorithmic procedure can generate different values of *G* and $FSR_{v}$ corresponding to the same ∆FSR or to very close values of ∆FSR, meaning that Vernier architectures with different RR lengths can exhibit very similar ∆FSR but different performance in terms of Vernier gain and overall FSR.

Practically, not all the calculated combinations can be taken into account for the device fabrication as their operation and sensing performance must be adapted to the experimental setup used for the optical characterization and to the optical readout, respectively. In particular, high-performance Vernier devices are usually characterized by very large overall $FSR_{v}$; the evidence of this behavior can be appreciated in Figure 4b where the Vernier devices with gains higher than ~200 exhibit overall Vernier transmittances with $FSR_{v}$ larger than 60 nm, thus resulting in only one overall Vernier peak spread over the entire wavelength range mentioned previously and characterized by very close thin resonances, whose detection is mainly limited by the optical resolution of the laser used in the experimental setup. The experimental demonstration of this concept can be seen in [12], where a Vernier gain as high as 150 has been achieved with a corresponding Vernier peak spread over a 100 nm-wide spectral range with a $FSR_{v}$ of roughly 100 nm. In this context, the optical readout, both intensity and wavelength interrogation based, can be quite challenging since short wavelength shifts cannot be appreciated in very wide spectral transmittances and large wavelength shifts might require a very wide spectral window to be tracked. On the contrary, by moving towards higher values of ∆FSR, Vernier configurations with high Vernier gains can exhibit shorter overall FSR, such as $FSR_{v}$ of about 35 nm or even lower than 10 nm, in this specific case study. However, as specified previously, the other aspect to be considered is the fact that the longer the cascade-coupled RR lengths the shorter the specific RR FSRs, which should be sufficiently larger to be detected by means of the experimental setup employed. For example, a wavelength resolution of 5 pm has been used for the accurate acquisition of the experimental data reported in this paper, *i.e.*, Vernier and single RR spectra with FSRs even lower than 0.5 nm. In conclusion, a total number of four Vernier architectures, labelled as A, B, C, and D and indicated in Figure 4a,b as well, have been selected among the 140 possible calculated combinations for the fabrication and experimental investigation.

**Figure 5:**
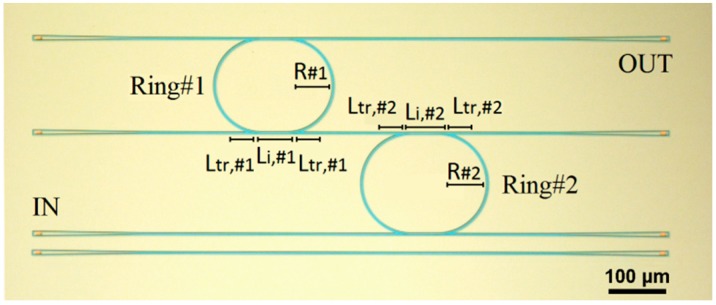
Optical image of a representative fabricated SOI Vernier device.

A micrograph of a representative Vernier device is plotted in Figure 5 with the fundamental geometrical dimensions labeled in. In particular, the cascade-coupled RRs are simply labeled as Ring#1 and Ring#2 with their radii indicated as $R_{\#1}$ and $R_{\#2}$, respectively. In this context, it is worth noting that the position of both RRs can be exchanged as the overall Vernier transmittance is the product of single RR transmittances and the commutative property is valid. Similarly, the input and output ports can be inverted as well without altering device operation and performance. However, such operating feature cannot be applied in the case of sensor arrays where many Vernier devices are cascaded to each other, as proposed in [12]. Finally, grating couplers have been used for coupling quasi-TE polarized light from fibers to the chip and *vice versa* and a reference waveguide, visible at the bottom of Figure 5, has been used for the normalization of the measured Vernier and RR spectra.

All the cascade-coupled RRs designed and fabricated in this investigation are characterized by symmetric DCs and this is the reason why their geometrical dimensions are labeled only on one side of each RR shown in Figure 5. In particular, $L_{i}$ is the interaction length where the distance between the coupled waveguides is constant and equal to *g_0_*, *i.e.*, the directional coupler gap (of the order of hundreds of nm, thus not visible in the micrograph). Furthermore, $L_{tr}$ is the transition length where *g_0_* is not constant and varies as a function of the particular arc-shaped bend waveguides of the RR. In particular, $L_{tr,\#i}$ is equal to ¾$\;R_{\#i}$ where *i* (*i.e*., 1 or 2) indicates the *i*-th RR of the Vernier architecture.

Referring to Figure 1, once the Vernier architectures have been selected for the design and fabrication, they are processed by means of the remaining blocks of the overall design flow, named as “Directional coupler design” and “Ring/racetrack resonator design”. In particular, these two blocks are arranged in a loop configuration because every variation in DC dimensions for achieving a desired power coupling coefficient ${\text{κ}}_{c}^{2}$, affects the RR design since the overall RR length $L_{\#i}$ is equal to 2π$R_{\#i}+2L_{i,\#i}$ with #*i* = #1 or #2, depending on the RR under investigation. Consequently, the design procedure can finish when the dimensions of both the symmetric DCs and RR lengths are set. To this purpose, a detailed theoretical and experimental investigation on DCs is reported in the following.

SOI based DCs, single RRs as well as cascade-coupled RRs based on the Vernier effect are very sensitive to waveguide fabrication tolerances [33]. Moreover, power coupling coefficients ${\text{κ}}_{c}^{2}$, which depend dramatically on geometrical dimensions of DCs, can also affect the operation of Vernier devices. Then, a sophisticated theoretical model based on the rigorous equations of the coupled mode theory (CMT) and super mode theory (SMT) already developed by us and described in [35], has been used for the design of DCs in order to achieve a very accurate control of power coupling coefficients. In particular, two types of DC devices have been taken into account: S-cosine DCs working as stand-alone devices and arc-shaped DCs for coupling light from a straight bus waveguide to a coupled RR in Vernier architectures.

**Figure 6:**
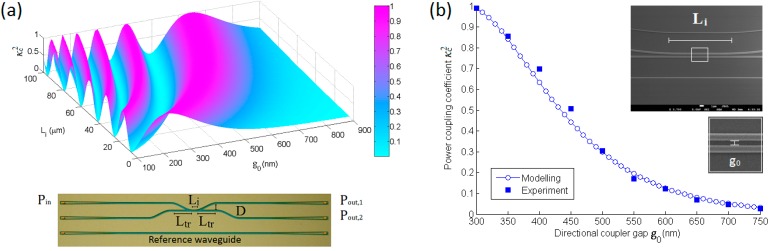
(**a**) 3D graph of ${\text{κ}}_{c}^{2}$ calculated as a function of $L_{i}$ and *g_0_* in a SOI rib-based S-cosine DC with the optical image of a representative S-cosine DC device ($L_{i}$ = 10 μm, $L_{tr}$ = 150 μm, *D* = 50 μm); (**b**) Power coupling coefficient, ${\text{κ}}_{c}^{2}$, as a function of different gaps, *g_0_*, of S-cosine DCs with dimensions: $L_{i}$ = 10 μm, $L_{tr}$ = 150 μm, *D* = 50 μm, and working at λ = 1.55 μm; Scanning Electron Microscope (SEM) image of the coupling region with a zoom of the DC gap are also shown.

By starting from DCs based on S-cosine bend SOI rib waveguides, an optical image of a representative fabricated device is shown in Figure 6a (bottom). In particular, $L_{i}$ is the interaction length where the distance between the coupled waveguides is constant and equal to *g_0_*, *i.e.*, the directional coupler gap. $L_{tr}$ is the transition length where *g_0_* is not constant and varies as a function of the particular S-cosine shape. Finally, the parameter *D* is the distance between the two DC arms at the end of both transition regions.

A 3D graph of the power coupling coefficient, ${\text{κ}}_{c}^{2}$, as a function of different values of $L_{i}$ and *g_0_*, has been calculated with $L_{tr}$ = 150 μm, *D* = 50 μm (Figure 6a, top). As expected by the rigorous formulation of the CMT, the 3D graph is characterized by a sinusoidal shape. In particular, the longer the interaction length and the smaller the DC gap *g_0_*, the shorter the distance between consecutive ${\text{κ}}_{c}^{2}$ peaks. In this specific case study, the control of the parameter ${\text{κ}}_{c}^{2}$ is much stronger, also against fabrication tolerances, when $L_{i}$ is shorter than 40 μm. Then, as a result of the design strategy proposed, DC dimensions can be arbitrarily selected in the 3D graph for achieving a desired value of ${\text{κ}}_{c}^{2}$ in the range of 0–1.

In order to proof experimentally the reliability and efficiency of our DC design procedure, we have fabricated a number of S-cosine DCs with dimensions: $L_{i}$ = 10 μm, $L_{tr}$ = 150 μm, *D* = 50 μm, and *g0* varied in the range from 300 nm to 750 nm in a 50 nm increment. In this way, ${\text{κ}}_{c}^{2}$ can be estimated accurately as the ratio $P_{out,2}/P_{in}$, where $P_{in}$ can be measured by means of the reference waveguide.

Experimental results plotted in Figure 6b evidence a very good agreement between measurements and theoretical predictions. Furthermore, the experimental methodology employed for the characterization of directional couplers has imposed independent measurements of each DC as a stand-alone device. In particular, with reference to the representative optical image plotted in Figure 6a, the output optical signals have been measured at the output ports (*i.e*., $P_{out,1}$, and$\;P_{out,2}$) of the coupled arms, while the optical signal at the input port, $P_{in}$, has been measured by means of the reference waveguide. Furthermore, it is worth noting that the consistency of the method has been verified systematically for each directional coupler by ensuring that the sum of the signal powers at the ports $P_{out,1}$ and$\;P_{out,2}$ was almost equal to $P_{in}$, in accordance with the principle of conservation of energy. Moreover, any possible fabrication non uniformity affecting the waveguides of the device under test can be found out by using this approach, because the methodology employed and based on the use of the reference waveguide for each DC has determined the measurement points plotted in Figure 6b, to be uncorrelated. Consequently, any possible fabrication errors would have affected each single point, resulting in a distinguishable discrepancy with respect to the overall power coupling coefficient trend.

The same design strategy implemented in the case of S-cosine DCs has been performed for the design of arc-shaped DCs used in Vernier devices. Furthermore, we have simulated the shifts of the resonant wavelengths of a single RR as well as the variation of the power coupling coefficient, $\kappa_{c}^{2}$, in arc-shaped DCs as a function of waveguide fabrication tolerances.

**Figure 7:**
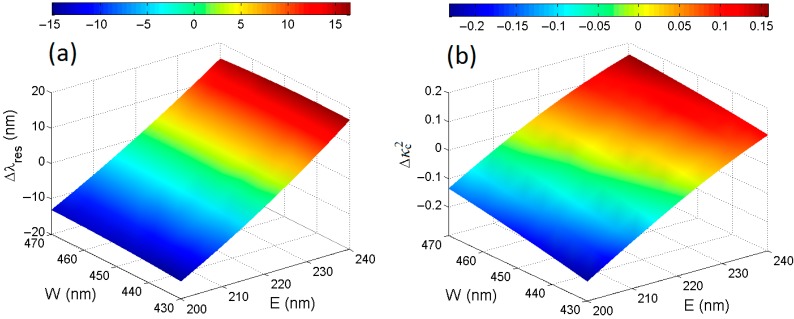
(**a**) Resonant wavelength and (**b**) power coupling coefficient variations (Δ${\text{λ}}_{res}$ and Δ${\text{κ}}_{c}^{2}$, respectively) as a function of SOI rib waveguide fabrication tolerances at the operating wavelength of λ = 1.55 μm.

Numerical results plotted in Figure 7a,b confirm the etch depth, *E*, as the most critical fabrication parameter because a maximum resonant wavelength shift, Δ${\text{λ}}_{res}=\;{\text{λ}}_{res}\left(W_{nom},E_{nom}\right)-{\text{λ}}_{res}(W,E)$ as large as ±~15 nm can occur, corresponding to a relative percentage shift of roughly ±1%. In addition, the power coupling coefficient variations Δ${\text{κ}}_{c}^{2}=\;{\text{κ}}_{c}^{2}\left(W_{nom},E_{nom}\right)-{\text{κ}}_{c}^{2}\left(W,E\right)$ can be as large as ±0.2 as shown in Figure 7b, where an arc-shaped DC with the nominal gap *g_0_* = 500 nm, $L_{i}$ = 10 μm, and $L_{tr}$ = 150 μm has been taken into account. Finally, it is worth specifying that the gap *g0* is not fixed in simulations but varies as a function of *W* in the range of $W_{nom}$ ± 20 nm, according to the equation: $g_{0}=\;g_{0,nom}+\;W_{nom}-W$ where $g_{0,nom}$ is the DC gap corresponding to the nominal waveguide dimensions.

In conclusion, the complete description of the selected Vernier configurations is reported in Table 2 where all the dimensions are listed, thus including RR lengths and radii as well as DC gaps, *g_0_* and interaction lengths $L_{i}$. The DC transition lenghts are not listed as they can be easily calculated as ¾$\;R_{\#i}$. Finally, it is worth noting that the symmetric DCs have been designed in order to exhibit power coupling coefficients as close to 0.1 as possible since these values of ${\text{κ}}_{c}^{2}$ have resulted to be suitable for achieving high extinction ratios (ER) in Vernier transmittances [34].

**Table 2:** Dimensions of fabricated SOI Vernier devices.

Device	$L_{\#1}$ (μm)	$L_{\#2}$ (μm)	$R_{\#1}$ (μm)	$R_{\#2}$ (μm)	$L_{i,\#1}$ (μm)	$L_{i,\#2}$ (μm)	*g_0_* (nm)
A	328.1	345.2	49	52	10.1	9.2	500
B	500.7	517.8	77	79	8.4	10.7	500
C	1117.3	1185.8	150	161	87.4	87	450
D	1204	1281.5	164	176	86.7	87.8	600

## 3. Test of the Vernier Device Operation and Performance

The efficiency and reliability of our strategies for the design of integrated DCs has been demonstrated by comparing simulation results with experimental measurements as shown in Figure 6b, where a very good agreement between measured and calculated values of ${\text{κ}}_{c}^{2}$ has been achieved. It is worth noting that S-cosine DCs has allowed a very accurate estimation of power coupling coefficients, which cannot be achieved by means of single or cascade-coupled RRs, where arc-shaped DCs have been used. Despite this, the influence of different power coupling coefficients on RR operation and performance has also been demonstrated by measuring normalized transmittances of the RR labeled as Ring#1 of Vernier B architecture, as a function of different values of the DC gap, *g_0_*, equal to 300, 500, 600 and 700 nm.

**Figure 8:**
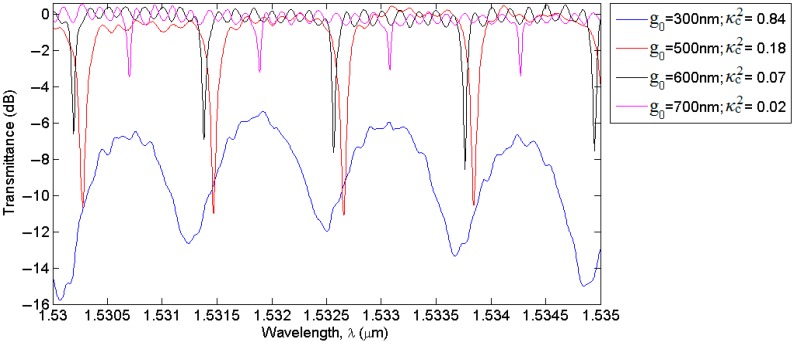
Influence of different values of arc-shaped DC gap, *g_0_*, on Vernier B Ring #1 transmittances.

Experimental results are plotted in Figure 8 in the spectral window extended from 1530 nm to 1535 nm. In particular, although the FSRs have resulted to be almost identical in the measured transmittances plotted on a decibel (dB) scale, experimental results evidence how insertion loss (IL) and ER vary dramatically as a function of ${\text{κ}}_{c}^{2}$ ranging from a minimum of 0.02 to a maximum of 0.84. Furthermore, it is clearly visible in Figure 8 that resonant peaks are positioned at slightly different wavelengths because of the process variability. However, a minimum resonant wavelength shift less than 1.5 Å and a maximum of ~5 Å occurred, thus corresponding to fabrication tolerances less than ±5 nm for the waveguide width and height, also according with the simulation results plotted in Figure 7a.

**Figure 9:**
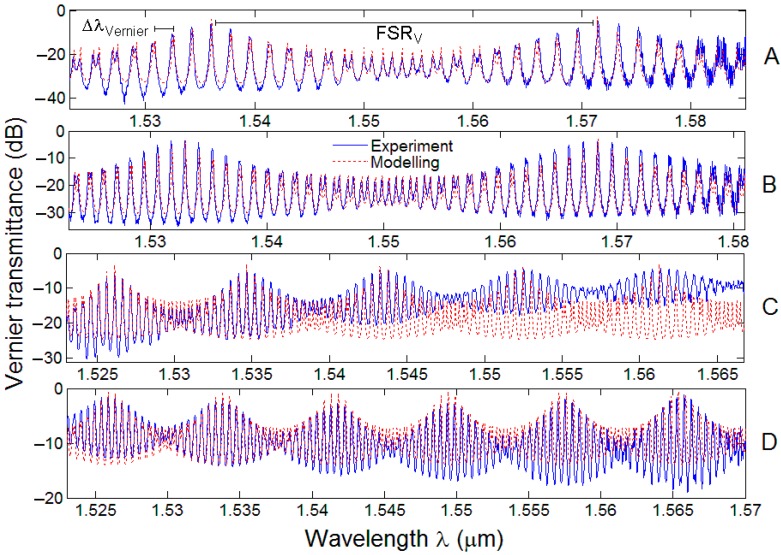
Normalized experimental transmittances (blue solid line) compared with theory (red dashed line) for Vernier A, B, C, and D architectures.

In conclusion, it is worth outlining that the accurate fabrication processes and facilities employed (*i.e*., the e-beam lithography and the ICP etching), have allowed to achieve a not-trivial waveguide parameter control. Normalized Vernier transmittances of the designed configurations labeled as A, B, C, D are plotted in Figure 9. Actually, the comparison between overall experimental and theoretical Vernier spectra confirms well the expected operation in the second Vernier regime in each device, where the Vernier envelope is made of a number of close resonances separated by a spectral distance, let us name it Δ${\text{λ}}_{Vernier}$.

As expected by our simulation results, spectral features of the normalized spectra change dramatically as a function of RR lengths. In fact, the longer $L_{\#1}$ and $L_{\#2}$ the shorter Δ${\text{λ}}_{Vernier}$, which is almost equal to the largest value between $FSR_{\#1}$ and $FSR_{\#2}$. Furthermore, it is possible to observe in Figure 9, that the number of Vernier peaks of the normalized spectra of A, C, and D configurations increased from two, to five and finally to six, respectively, in the measured spectral range of 1520–1580 nm. The optical parameters characterizing the Vernier devices, such as the overall Vernier FSR, $FSR_{v}$, the power coupling coefficients ${\text{κ}}_{c,\#1}^{2}$, ${\text{κ}}_{c,\#2}^{2}$, the maximum extinction ratios ($ER_{max}$) measured at the central resonance peak of the highest overall Vernier peak, and the average insertion losses ($IL_{avg}$), are listed in Table 3.

**Table 3:** Experimental optical parameters of fabricated SOI Vernier devices.

Device	$IL_{avg}$ (dB)	$ER_{max}$ (dB)	$FSR_{\#1}$ (nm)	$FSR_{\#2}$ (nm)	ΔFSR (nm)	$\kappa_{c,\#1}^{2}$ ±0.05	$\kappa_{c,\#2}^{2}$ ±0.05
A	5.7	29.6	1.8531	1.7613	0.0918	0.13	0.12
B	3.7	30.1	1.2167	1.1765	0.0400	0.16	0.21
C	4.9	23.5	0.5449	0.5134	0.0315	0.16	0.16
D	2.1	17.7	0.5095	0.4784	0.0306	0.31	0.27

In all the spectra plotted in Figure 9, the shape of the resonances constituting the Vernier peaks is not constant throughout the overall experimental wavelength range, especially for Vernier configurations with long roundtrip lengths. This effect caused the ER not to remain constant as a function of the operating wavelength, although IL is always stable. The reasons for this behavior are due to both wavelength dependence of input/output gratings and mainly chromatic dispersion that caused very short shifts (*i.e*., ~pm) of RR resonances as well as changes of DC power coupling coefficients, ${\text{κ}}_{c}^{2}$, for DCs of each Vernier device. In fact, the slope coefficient of ${\text{κ}}_{c}^{2}$ as a function of the operating wavelength has been estimated to be ~1.2 × 10^−3^ nm^−1^.

**Figure 10:**
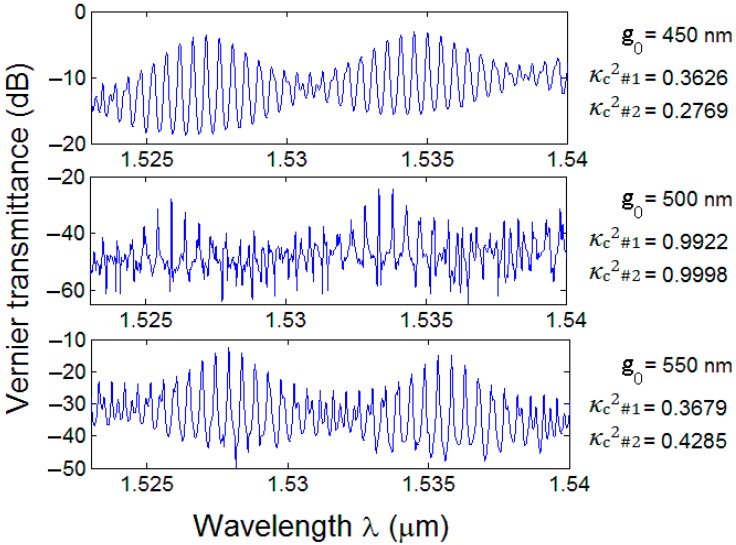
Normalized transmittances of Vernier D architecture as a function of different directional coupler gap values with estimated power coupling coefficients.

The configuration labeled as Vernier D has been fabricated with the same dimensions as listed in Table 2, varying only *g_0_* from 450 nm to 550 nm in a 50 nm increment. As shown in Figure 10, power coupling coefficients (*i.e*., ${\text{κ}}_{c,\#1}^{2}$, ${\text{κ}}_{c,\#2}^{2}$) of DCs of both cascade-coupled RRs affect dramatically the overall shape of the Vernier spectra as well as ER and IL of each Vernier device. In particular, it is worth specifying that the irregular spectral response measured with *g_0_* = 500 nm is not an artifact of the measurement, since all the spectra of Figure 10 have been measured by using identical setup conditions (*i.e*., fiber alignment, spectral resolution, output laser power). Furthermore, such behavior is also justified qualitatively by the simulated plot shown in Figure 6a, where the power coupling coefficient has been calculated as a function of the DC interaction length, $L_{i}$, and gap, *g_0_*. Actually, although the aforementioned plot referes to an S-cosine DC, simulation results have shown a very similar behavior in the case of arc-shaped DCs. In particular, it is evident that the longer the interaction length the less tolerant ${\text{κ}}_{c}^{2}$ against DC gap variations, depending also by the dimensions of the fabricated waveguides. On the contrary, ${\text{κ}}_{c}^{2}$ varies smoothly as a function of *g_0_* and is more tolerant against fabrication tolerances when short interaction lenghts characterize the DCs. Indeed, a demonstration of this can be appreciated in Figure 8 where ${\text{κ}}_{c}^{2}$ decreases as a function of the increasing DC gap, exhibiting a monotonically decrescent path and interaction lengths as short as 8.4 µm and 10.7 µm characterize Vernier B RRs, thus being much shorter than 86.7 µm and 87.8 µm of DCs of Vernier D device in Figure 10.

The influence of process variability is also evident in Figure 10, where Vernier peaks are centered at slightly different wavelengths (*i.e*., Δ${\text{λ}}_{peak}$ < 2 nm), thus confirming waveguide fabrication tolerance in the range of ±5 nm. The design and fabrication parameter control has been proven by comparing experimental Vernier FSRs and gains, *i.e.*, $FSR_{v,exp}$ and $G_{exp}$, respectively, with those calculated by our modelling. Then, relative percentage errors of the Vernier FSR defined as ${\text{ε}}_{r,FSR_{v}}=\left(\left(FSR_{v}-FSR_{v,exp}\right)/FSR_{v}\right)\times 100$, have been calculated and listed in Table 4. Analogously, Vernier gain errors, ${\text{ε}}_{r,G}$, have been calculated by substituting *G* to $FSR_{v}$ and $G_{exp}$ to $FSR_{v,exp}$ in the expression reported previously.

Finally, relative errors ${\text{ε}}_{r,FSR_{v}}$ and ${\text{ε}}_{r,G}$ lower than 1.2% and 0.8% have been achieved, respectively, in each of the fabricated Vernier devices, confirming again the rigorous and accurate design and fabrication parameter control as well as the reproducibility of the implemented design flow. A further demonstration of this very good agreement between experiments and theoretical predictions can be appreciated in Figure 4a,b, where circles referring to each fabricated device do not only indicate the corresponding Vernier gains and FSRs, but also the experimental values measured from the specific device characterization.

**Table 4:** Relative errors of FSRs and gains in the fabricated SOI Vernier devices.

Device	$FSR_{v}$ (nm)	$FSR_{v,exp}$ (nm)	$\varepsilon_{r,FSR_{v}}$ %	G	$G_{exp}$	$\varepsilon_{r,G}$ %
A	35.86	35.50	1.00	20.18	20.20	−0.09
B	35.86	35.61	0.69	30.28	30.30	−0.06
C	9.00	8.89	1.22	17.42	17.30	0.68
D	7.91	7.84	0.88	16.53	16.40	0.78

Actually, our most performant device, *i.e.*, the configuration Vernier B with a gain as high as ~30, can compete with similar Vernier sensors demonstrated experimentally with gains of 24.3 (ΔFSR = 27 pm) [10], 27.2 (ΔFSR = 80 pm) [11], and 26.8 (ΔFSR = 8.2 pm) [13], to mention a few. In this context, it is worth specifying that the devices designed and fabricated in this investigation have not been optimized for RI sensing. In fact, SOI rib waveguides used here are characterized by an homogeneous sensitivity for the fundamental quasi-TE optical mode as low as $S_{h}$ ≈ 0.04, thus resulting in estimated theoretical performance of the Vernier device B equal to $S_{\text{λ}}$ = 479 nm/RIU and LOD = 2.5 × 10−3 RIU. For example, by considering a SOI slot waveguide with an homogeneous sensitivity, $S_{h}$, almost equal to 1 instead of the rib waveguides, numerical results revealed a huge enhancement, resulting in $S_{\text{λ}}$ = 11,496 nm/RIU and a LOD = 1 × 10^−4^ RIU. Consequently, a very accurate design of optimized Vernier devices for RI sensing applications must be carried out in order to achieve ultra-high performance by means of the accurate and reproducible design procedure proposed.

The design procedure proposed here has been successfully performed for the design and fabrication of Vernier devices based on SOI cascade-coupled RRs operating for the first time in the MIR wavelength range of 3.7–3.9 µm, so opening intriguing scenarios in MIR photonic sensing applications. In particular, a number of Vernier configurations based on SOI rib waveguides have been demonstrated experimentally with Vernier gains of 19.40 and 18.87 corresponding to ΔFSR of 270 pm and 740 pm and $FSR_{v}$ of 98 nm and 249 nm, respectively [41]. A further demonstration of the reliability and reproducibility of the implemented design flow is demonstrated by the experimental characterization of Vernier devices based on SOI fully-etched waveguides operating in the same MIR wavelength range as before. In particular, Vernier gains of 19.94 and 18.12 have been achieved, corresponding to Vernier FSRs of 71.81 and 99.32 and ΔFSR of 190 pm and 320 nm, respectively [29]. In conclusion, the aforementioned Vernier devices have also been used to demonstrate sensing in the MIR. In fact, perfluorodecalin, which exhibits low absorption around 3.8 µm, has been concentrated on top of the SOI chip by means of a PDMS microfluidic channel and an overall Vernier wavelength shift$\;∆{\text{λ}}_{v}=∆{\text{λ}}_{sensor}$ ≈ 38 nm has resulted due to a cover RI change of $∆n_{c}$ ≈ 10−1 RIU [42]. Actually, the tested Vernier device was not covered by an insulating layer, thus both cascade-coupled RRs were exposed to the same cover medium and shifted analogously. Consequently, the physical Vernier gain can be estimated as 1, resulting in $∆{\text{λ}}_{v}=∆{\text{λ}}_{sensor}$ as reported above. Practically, the expected Vernier wavelength shift, assuming the overall chip with an insulating layer on top and an opened window on one of the two cascade-coupled RRs, can be as large as $∆{\text{λ}}_{v}$ = 757.72 nm and $∆{\text{λ}}_{v}$ = 688.56 nm corresponding to Vernier gains *G* of 19.94 and 18.12, respectively, thus revealing the huge enhancement of sensing performance achievable by means of the Vernier effect.

## 4. Conclusions

In this paper, the experimental application of a special design flow for Vernier-based photonic sensors as well as a very accurate control of the design and fabrication parameters in SOI cascade-coupled RRs working in the second regime of the Vernier effect, have been demonstrated in the NIR wavelength range of 1520–1580 nm. The influence of cascaded RR lengths, DC dimensions, waveguide width and etch-depth as well as fabrication tolerances on Vernier device operation and performance has been demonstrated by means of a detalied investigation which, to the best of our knowledge, has never been reported in literature. Actually, the most important figures of merit of our Vernier devices, such as the Vernier gain *G*, the spectral distance ${\text{Δλ}}_{Vernier}$, and the overall Vernier FSR, $FSR_{v}$, have been analyzed experimentally and compared with the theoretical predictions, achieving minimum relative errors even lower than 1%. In particular, Vernier gains as high as 30, minimum and maximum overall Vernier FSRs of ~8 nm and ~35 nm have been demonstrated with insertion losses ranging from 2.1 dB up to 5.7 dB, which are comparable to state-of-the-art device performance [10,11,12,13,14,15,17,18]. Furthermore, it is worth noting that some of the devices proposed here have also been used for the efficient demonstration of an innovative device-level experimental tool for the characterization of the flow of light in integrated photonic circuits using ultrafast photomodulation spectroscopy [43]. In addition, they have been tested as picosecond optically reconfigurable filters by means of all-optical photomodulation, resulting in phase-shifts even larger than 2π [44].

We have demonstrated that the implemented design flow based on sophisticated algorithmic routines and rigorous theoretical modelling can be used systematically for the reliable and reproducible design of Vernier devices fabricated on the conventional SOI technology platform by means of standard e-beam lithography and ICP etching. Indeed, four different Vernier architectures have been fabricated and tested, working properly after the first fabrication run and very close to the predicted performance. Furthermore, different wavelength ranges and integrated waveguides can also be explored as demonstrated by the operation of Vernier devices based on SOI rib and strip waveguides at the intriguing MIR wavelength range, where the optical absoprtion can be used with the conventional RI sensing principles, *i.e.*, the homogeneous and surface sensing [29,41,42].

The design flow proposed in this paper allows to take into account fabrication tolerances imposed by the foundry and technology platform employed as well as a number of crucial parameters which are worth being processed simultaneously in the design of such Vernier architectures and optimized as a function of the specie to be sensed [34]. For example, geometrical and optical properties of integrated waveguides, directional couplers and ring/racetrack resonators as well as the chromatic dispersion and the operating wavelength range, are worth being mentioned among all. Finally, the flexibility and reliability of the overall design tool can be easily extended to the design of Vernier sensors also based on fiber optic technology [45,46], or on other group IV technology platforms such as germanium-on-silicon, germanium-on-SOI, and to Vernier-based lab-on-a-chip sensing platforms with mixed combinations of cascade-coupled integrated photonic devices, e.g., MZIs and RRs suitable for ultra-high performance label-free chemical and biochemical sensing.
